# Genotypic Comparison of *Pasteurella multocida* from Healthy Animals at Entry to the Feedlots with That and from Bovine Respiratory Disease-Affected Animals during the Fattening Period

**DOI:** 10.3390/ani13172687

**Published:** 2023-08-22

**Authors:** Johan Manuel Calderón Bernal, Carlos Serna, Ángel García Muñoz, Alberto Díez Guerrier, Lucas Domínguez, José Francisco Fernández-Garayzábal, Ana Isabel Vela, Dolores Cid

**Affiliations:** 1 Departamento de Sanidad Animal, Facultad de Veterinaria, Universidad Complutense, 28040 Madrid, Spain; johancal@ucm.es (J.M.C.B.); carlsern@ucm.es (C.S.); aadiez@ucm.es (A.D.G.); lucasdo@ucm.es (L.D.); avela@ucm.es (A.I.V.); lcid@ucm.es (D.C.); 2 Departamento Producción y Sanidad Animal, Salud Pública Veterinaria y Ciencia y Tecnología de los Alimentos, Facultad de Veterinaria, Universidad Cardenal Herrera-CEU, CEU Universities, 46115 Valencia, Spain; angel@uchceu.es; 3 Centro de Vigilancia Sanitaria Veterinaria (VISAVET), Universidad Complutense, 28040 Madrid, Spain

**Keywords:** *Pasteurella multocida*, genotyping, apparently healthy animals, BRD-diseased animals

## Abstract

**Simple Summary:**

*Pasteurella multocida* can be found as a commensal resident in the nasopharynx of healthy calves but is considered one of the major bacterial pathogens contributing to bovine respiratory disease (BRD). We compared the genetic characteristics of *P. multocida* recovered from apparently healthy animals (*n* = 74) at the time of entry to the feedlot with isolates from animals affected by BRD during the fattening period (*n* = 32) using different molecular techniques (capsular and LPS typing, as well as PFGE). The genomes of a subset of 14 *P. multocida* isolates identified in clinically and non-clinically affected calves were sequenced, and in silico analysis of multilocus sequence types, virulence-associated genes, and antimicrobial resistance genes was carried out. These isolates of *P. multocida* belonged mainly to sequence types ST79 and ST13, but ST80 was also identified. Isolates were further compared with the genome sequences available in the NCBI genome database of 53 *P. multocida* isolates of ST13, ST79, and ST80 from different geographical locations for a global comparison.

**Abstract:**

The aim of this study was to investigate the possible genotypic differences between commensal *Pasteurella multocida* isolates from apparently healthy animals (AHA) at the time of entry to feedlots and those from BRD-affected animals (BRD-AA). A total of 20 batches of beef calves in seven feedlots were followed-up during the fattening period. *P. multocida* was isolated from 28.1% of AHA and 22.9% of BRD-AA. All isolates belonged to the A: L3 genotype. Most isolates from clinical cases (81.0%) grouped into a PFGE cluster were significantly associated with BRD cases (OR, 24.9; 95% CI, 6.4–96.2). The whole genomes of 14 isolates representative of the pulsotypes most frequently detected in BRD-AA and AHA were sequenced and compared with 53 bovine genomes belonging to the identified ST13, ST79, and ST80 genotypes for a global comparison. No differences were found in the virulence-associated gene content between sequence types (STs) globally or between BRD-AA and AHA isolates in this study. Significantly, ST79 isolates harbored ARGs, conferring resistance to different antimicrobials, including macrolides and tetracyclines, which are commonly used for the treatment of BRD. Two Spanish ST79 isolates carried an ICE highly similar to ICE Tn*7407*, which was recently detected in Germany, suggesting that ST79 *P. multocida* isolates in Europe and North America may be associated with different ICEs.

## 1. Introduction

*Pasteurella multocida* is one of the main bacterial pathogens contributing to bovine respiratory disease (BRD) [1,2,3,4,5]. *P. multocida* can be found as a commensal resident in the nasopharynx of healthy animals and has been isolated from both healthy and sick cattle [2,6,7]. In the etiology of BRD, *P. multocida* is considered an opportunistic pathogen able to reach the lungs and cause disease after exposure to different risk factors, including respiratory viral infections and management and environmental stress factors [1,6]. 

*P. multocida* is a genetically heterogeneous bacterium causing diverse diseases in a variety of domestic and wild animals and occasionally in humans [8]. In epidemiological studies, classical serological classification into 5 serogroups (A, B, C, E, and F) and 16 serotypes based on capsular polysaccharide and lipopolysaccharide (LPS) antigens, respectively, has been substituted by PCR classification into five capsular types and eight (L1–L8) LPS genotypes [9,10]. Moreover, other virulence-associated factors have been described in *P. multocida*, such as fimbriae and other adhesins, PMT toxins, iron acquisition proteins, sialic acid metabolism, hyaluronidase, outer membrane proteins (OMPs), and superoxide dismutase [8]. Although the molecular basis for host or disease specificity has not been found, epidemiological data and molecular typing studies have indicated that some genotypes are associated with certain hosts or diseases [8,11,12,13,14]. 

*P. multocida* BRD-associated isolates have shown a very low level of genetic diversity, as demonstrated in several studies using a variety of typing methods, including capsular and LPS genotyping, virulotyping, and multilocus sequence typing (MLST) [5,6,13]. *P. multocida* isolates from BRD clinical cases belong mainly to the A: L3 genotype and closely related sequence types ST79, ST13, or ST80, which are included in clonal complex CC13 [5,6]. Sequence types ST79 and ST13 also seem to be the most prevalent among respiratory isolates from healthy cattle [6]. Whole-genome sequencing with phylogenetic analysis of *P. multocida* genomes identified ST79 as the most dominant clonal lineage in BRD-associated isolates worldwide and suggested that ST79 shows host specificity to cattle but genomic heterogeneity [8,14,15]. Using PFGE, it was found that some closely related clones (pulsotypes) were consistently associated with clinical cases of BRD [5]. However, whether these clones are prevalent among healthy animals is unknown, since studies analyzing the molecular characteristics of *P. multocida* isolates from healthy animals are scarce [6]. The aim of this study was to investigate whether there are genotypic differences between commensal *P. multocida* isolates from apparently healthy animals at the time of entry to the feedlots and those from animals that developed BRD during the fattening period in the same feedlot batches.

## 2. Materials and Methods

### 2.1. Ethics Statement

Research procedures were carried out in accordance with national and institutional regulations, and the project was approved by the Research Ethics and Biosafety Committees of Universidad Complutense de Madrid (CB_20230215-03_SAL).

### 2.2. Animal Sampling

A total of 20 batches of crossbred beef calves (5–7 months old) were followed-up during the fattening period (6–7 months) to identify animals with clinical signs compatible with BRD, defined as animals with nasal or ocular discharges, spontaneous cough, difficult breathing, or a rectal temperature of over 40 °C. The batches entered the fattening period from 18 January 2021 to 20 September 2022 in seven different feedlots (Table 1). A total of 12 batches (LV1–LV12) of 80–87 animals each were from a large feedlot (>15,000 animals fattened-up per year) located in Valencia (Spain), and 8 batches (LM1–LM8) of 15–30 animals each were from 6 small feedlots (<200 animals fattened-up per year) located in the central region of Spain. All the batches in the large feedlot (LV1–LV12) and in one small feedlot (LM5) included animals of multiple origins that were previously purchased and mixed by commercial intermediary operators, whereas the batches from the remaining small feedlots included animals born on their respective farms (Table 1). Respiratory samples from 83 animals that developed clinical signs compatible with BRD during the follow-up period were collected on the first day of detection of clinical signs and prior to antimicrobial treatment with deep nasopharyngeal swabs (DNPS, Dryswab laryngeal MW128, MWE Medical Wire, Corsham, UK; *n* = 59) or bronchoalveolar lavage (BAL; *n* = 24). BAL was collected by a sterile catheter using a commercial BAL sampling pack (Exopol, Zaragoza, Spain). Briefly, the procedure was performed in standing animals without sedation by inserting the catheter medioventrally through the nasal cavity, larynx, and trachea until the bronchi, following nostril cleaning and disinfection with 90% alcohol. A total of 40 mL of sterile PBS was injected through the catheter and immediately aspirated; then, the fluid was dispensed in sterile containers.

In addition, a convenience sample of 178 apparently healthy animals in a subset of 12 batches (*n* = 6 in the large feedlot and *n* = 6 in the small feedlots), including up to 20 animals per batch in the large feedlot (*n* = 119) and at least 5 animals per batch in the small feedlots (*n* = 59), was sampled on the second day after entry to the feedlot in order to detect *P. multocida* carriage. Respiratory samples from apparently healthy animals were collected by DNPS (Dryswab laryngeal MW128, MWE Medical Wire, Corsham, UK). On the same day, the animals in all the batches were vaccinated with commercial polyclostridial toxoid vaccines and BRD vaccines against bovine parainfluenza 3 virus, bovine respiratory syncytial virus, bovine viral diarrhea virus, and *Mannheimia haemolytica* A1 and revaccinated 3–4 weeks later. A dose of ivermectin was also administered with the first dose of vaccines.

### 2.3. P. multocida Isolation and Identification

DNPS and BAL samples (150 µL) were inoculated on Columbia agar supplemented with 5% sheep blood (BioMérieux España, Madrid, Spain) and incubated aerobically at 37 °C for 24 h. Colonies with macroscopic characteristics compatible with *P. multocida* were identified by matrix-assisted laser desorption/ionization mass spectrometry (MALDI-TOF MS) (Bruker Daltonik GmbH, Germany) as described by Pérez-Sancho et al. [16]. A PCR assay targeting the *kmt1* gene, which is species-specific to *P. multocida*, was used to confirm the identification of the isolate using to the primers and the conditions defined by Townsend et al. [17]. Bacteria were stored at −40 °C until use.

### 2.4. Capsular, Lipopolysaccharide, and Pulsed-Field Gel Electrophoresis (PFGE) Typing of P. multocida Isolates

All *P. multocida* isolates (*n* = 106; 32 from cases and 74 from apparently healthy animals) were subjected to capsular and LPS detection by PCR. Bacterial DNA was obtained as described by Calderón et al. [5]. Capsular types (A, B, D, E, and F) were determined by a multiplex PCR, as described by Townsend et al. [9]. LPS genotypes (L1–L8) were identified by multiplex PCR, as described by Harper et al. [10]. All the primers were synthesized by STAB Vida laboratories (Caparica, Portugal). *P. multocida* strains NCTC 10322 (*cap-A*), NCTC 10323 (*cap-B*), NCTC 12178 (*cap-D* and *lps-6*), NCTC 10326 (*cap-E* and *lps-2*), and C104013 (*cap-F* and *lps-3*) were used as positive controls for the indicated genes.

All *P. multocida* isolates, except four isolates that did not grow after freezing, were subjected to PFGE as previously described [5]. *Salmonella enterica* serovar Braenderup H9812 restricted with enzyme *XbaI* (Thermo Fisher, Madrid, Spain) was used as a molecular weight control. Similarities between PFGE profiles were analyzed through visual comparison of band patterns using BioNumerics v8.1.1 software (Applied Maths, Sint-Martens-Latem, Belgium). *P. multocida* strains P21 and P153 of pulsotypes A and B, respectively, determined in a previous study [5], were included in the PFGE analysis for comparison.

### 2.5. Whole-Genome Sequencing (WGS), Assembly, and Multilocus Sequence Typing

**Figure 1:**
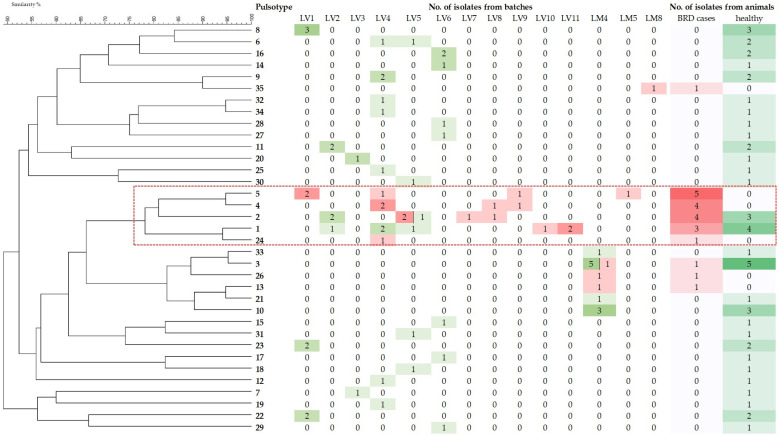
Dendrogram based on UPGMA cluster analysis displaying the level of similarity among the 69 respiratory bovine *P. multocida* isolates—21 from BRD-affected animals and 48 from apparently healthy animals at the time of entry to the feedlot following *Bsp120I* endonuclease DNA digestion—and heat map showing their distribution by batch and health status. The color scale indicates the number of isolates in each PFGE pattern from BRD cases (red) and from healthy animals (green). The red dashed rectangle indicates cluster I, which was significantly associated with BRD cases (OR: 24.9; 95% CI: 6.4–96.2).

In total, 14 isolates belonging to the pulsotypes most frequently detected in clinically affected animals (*n* = 6) and apparently healthy animals (*n* = 8) from different batches were selected for WGS (see Section 3, Figure 1). Genomic DNA was extracted using the Maxwell® Prokaryote/Eukaryote SEV DNA Purification Kit (Madison, WI, USA) protocol. DNA concentration was determined using a Qubit® fluorometer (Invitrogen; Waltham, MA, USA). DNA libraries were prepared using a Nextera XT DNA Library Preparation Kit (Illumina Inc., San Diego, CA, USA). Paired-end sequencing (2 × 150 bp) was carried out using the Illumina NovaSeq 6000 system at Macrogen Europe (Amsterdam, the Netherlands). After quality control and preprocessing, Illumina short reads were assembled with Shovill v1.1.0 software using default parameters (https://github.com/tseemann/shovill (accessed on 1 June 2023)). Genome assembly quality was assessed with QUAST v5.0.2 [18]. Bakta v1.4.0 software [19] was used to annotate the draft genome sequences of the 14 bovine *P. multocida* isolates. 

In silico multilocus sequence typing (MLST) was carried out to determine the sequence type (ST) of the 14 bovine *P. multocida* genomes sequenced in this study. In addition, MLST was carried out in all publicly available genome sequences of *P. multocida* from bovine hosts from different countries included in the National Center for Biotechnology Information (NCBI) Pathogen Detection database (https://www.ncbi.nlm.nih.gov/pathogens/) on 16 May 2023 to select bovine genomes belonging to sequence types ST13, ST79, and ST80 identified in this study (see Section 3). MLST was performed using the MLST v2.10 tool (https://github.com/tseemann/mlst), scanning contig files against the RIRDC MLST scheme [20]. The 53 bovine genomes from the NCBI database identified as ST13, ST79, and ST80 and the 14 genomes sequenced in this study (Table S1) were screened for the detection of virulence-associated and antimicrobial resistance genes, mobile genetic elements, and phylogenetic analysis.

### 2.6. Detection of Virulence-Associated Genes, Antimicrobial Resistance Genes, and Mobile Genetic Elements

The presence of 27 virulence-associated genes (VAGs; Table S2) encoding fimbriae and other adhesins (*ptfA*, *fimA*, *hsf1*, *hsf2*, *flp1*, *pfhA*, *pfhB1*, and *tadD*), toxins (*toxA*), iron acquisition proteins (*exbB*, *exbD*, *hgbA*, *hgbB*, *fur*, *tbpA, tbpB*, and *tonB*), sialic acid metabolisms (*nanB* and *nanH*), hyaluronidase (*pmHAS*), outer membrane proteins (OMPs) (*ompA*, *ompH*, *oma87*, *plpE*, and *plpB*), and superoxide dismutases (*sodA* and *sodC*) previously detected in bovine *P. multocida* isolates [5,11,14,21,22] was investigated in the 67 bovine genome assemblies (Table S1) using ABRicate v1.0.1 software (https://github.com/tseemann/abricate (accessed on 10 June 2023)). Sequences producing alignments with a minimum of 80% similarity and 90% coverage were considered to contain the virulence factor. The presence of acquired antimicrobial resistance genes was evaluated in the *P. multocida* genome assemblies using ABRicate v1.0.1 with the ResFinder database [23], applying the same criteria. 

To assess the presence of potential mobile genetic elements, genome assemblies were screened against the PlasmidFinder database [24] using ABRicate v1.0.1 to identify plasmid replicons. Additionally, a BLAST search was conducted using the I*CEPmu1* sequence from bovine *P. multocida* 36950 (CP003022) [25] to detect this integrative and conjugative element. Genome assemblies that aligned with the *ICEPmu1* reference sequence with at least 50% coverage and 90% identity were categorized as containing ICE. For two isolates from this study in which a putative ICE was detected (see Section 3), similarity to ICE Tn*7407*, which was previously described from a German isolate (GenBank accession no. CP087380; [26]) was assessed. Illumina short reads were mapped against Tn*7407* using the BWA v0.7.17 tool [27]. Subsequently, the coverage and percentage identity were calculated using SAMtools flagstat v1.3.1 (http://www.htslib.org/ (accessed on 15 June 2023)).

### 2.7. Phylogenetic Analysis

To assess the phylogenetic relationships between the 14 *P. multocida* genomes considered in this study and globally between the 67 bovine *P. multocida* genomes belonging to ST13, ST79, and ST80, whole-genome single-nucleotide polymorphism (WG-SNP) alignments were generated using Snippy v4.6.0 software (https://github.com/tseemann/snippy (accessed on 10 June 2023)) with the *P. multocida* 14424 ST79 genome (CP029322.1) as the reference. The starting phylogenetic tree was inferred with IQ-tree v2.2.0 [28] using the K3P+ASC or TVM+F+I+I+R3 model, selected on basis of the Bayesian information criterion (BIC). To account for recombination events and correct branch lengths in the starting tree, ClonalFrameML v1.12 [29] was employed. The total number of single SNPs in the filtered whole-genome sequence alignment was extracted using SNP-sites v2.5.1 [30]. R package ggtree v3.4.0 [31] and Inkscape v1.2.1 (https://inkscape.org/ (accessed on 10 June 2023)) were used to visualize and annotate the phylogenetic trees with metadata columns.

### 2.8. Data Analysis

Genetic diversity (GD) was estimated as the ratio between PFGE patterns and the number of isolates [5]. Isolates with identical pulsotypes from the same animal and date were considered to be the same strain, and only one isolate was included to assess the genetic diversity of isolates from clinical cases and healthy animals. Associations between categorical variables (PFGE cluster I, health status and animal source) were analyzed with Fischer’s exact test, and odds ratios (ORs) an their 95% confidence intervals (95% CIs) were calculated using Epi-InfoTM v7.2.5 software of the Center for Disease Control and Prevention (CDC) (http://wwwn.cdc.gov/ (accessed on 25 May 2023)). The significance level was set at *p* < 0.05.

## 3. Results

### 3.1. P. multocida Capsular-LPS Genotypes in Apparently Healthy and Clinically Affected Animals

*P. multocida* was detected in 50 of the 178 (28.1% ± 6.5%) apparently healthy animals sampled at the time of entry to the feedlot and in 19 of the 83 (22.9% ± 4.6%) clinically affected animals detected during the fattening period. There were no significant differences in these frequencies (OR: 1.3; IC 95% CI: 0.7–2.4; *p* < 0.05). A total of 106 *P. multocida* isolates were obtained: 74 from the 50 healthy animals and 32 from the 19 clinically affected animals that were positive for *P. multocida*. All the isolates belonged to the A: L3 genotype.

### 3.2. PFGE Typing

A single isolate was identified in 36 animals, while more than one isolate was obtained from 29 animals. In 25 of these animals, the isolates exhibited identical pulsotypes and according to the criterion that isolates with identical pulsotypes from the same animal and date be considered the same strain, only one isolate per animal was considered to calculate the GD (*n* = 25). In the remaining four animals, isolates with different pulsotypes were isolated from each animal and were therefore considered different strains (*n* = 8 isolates). Thus, overall, 69 isolates (48 from healthy animals and 21 from clinical cases) were included in GD analysis, which yielded 35 different PFGE patterns (Figure 1). In total, 29 pulsotypes were detected in the isolates from healthy animals (GD = 0.60), whereas 9 pulsotypes were detected in the clinical isolates (GD = 0.43). Most isolates from the clinical cases (81.0%) grouped into a cluster (cluster I) that included pulsotypes 1, 2, 4, 5, and 24 (Figure 1). This cluster was significantly associated (*p* < 0.05) with the health status (81.0 % from animals affected with BRD vs. 14.6 % from apparently healthy animals; OR: 24.9; 95% CI: 6.4–96.2) and the origin of animals (43.6% from batches that included purchased animals from different origins vs. 0% from batches that included animals originating from the respective farms). Moreover, isolates of cluster I were widely distributed, as they were detected in eight batches (LV1, LV4, LV5, and LV7–LV11) of the large feedlot farm (and one batch of a small feedlot (LM5; Figure 1). All of them included purchased animals of different origins (Table 1). Isolates of cluster I were not detected in batches that included animals originating from their respective farms (LM4 and LM8; Figure 1 and Table 1).

### 3.3. MLST Analysis and Screening of Virulence-Associated and Antimicrobial Resistance Genes and Mobile Genetic Elements in the Most Frequently Detected P. multocida Pulsotypes

**Figure 2:**
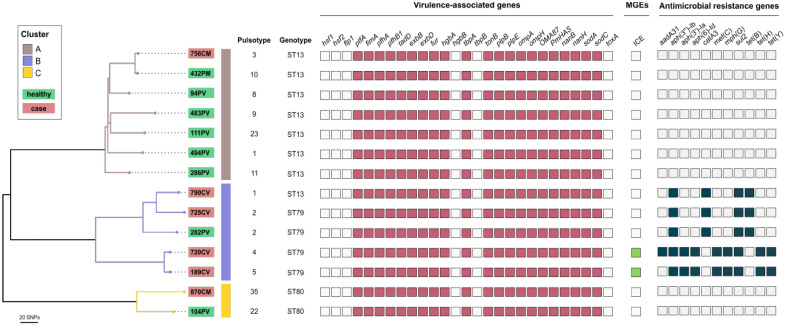
Phylogenetic tree generated on the basis of whole-genome SNP (WG-SNP) analysis of 14 bovine *P. multocida* isolates representative of the most frequently detected PFGE restriction patterns found in this study in BRD cases (*n* = 6) and apparently healthy animals at the time of entry to the feedlot (*n* = 8). The figure also shows the in silico identification of sequence type (ST), virulence-associated genes, mobile genetic elements (MGEs), and antimicrobial resistance genes.

WG-SNP phylogenetic analysis of the genomes of 14 *P. multocida* isolates representative of the pulsotypes most frequently detected among the clinical cases (*n* = 6) and healthy animals (*n* = 8) identified three distinct clusters (A–C; Figure 2). Cluster B included mainly clinical isolates (*n* = 4) and one isolate from a healthy animal, all belonging to pulsotypes of the PFGE cluster I (pulsotypes 1, 2, 4, and 5). The clinical isolates belonged to the ST79 (*n* = 3) and ST13 (*n* = 1) genotypes. The single isolate from a healthy animal belonged to the ST79 genotype. Cluster A contained seven isolates, all assigned to ST13, and the majority (*n* = 6) were from healthy animals. Cluster C consisted of two isolates: one from an animal affected by BRD and one from an apparently healthy animal, both assigned ST80 (Figure 2). 

No differences in the detection of virulence-associated genes were observed among isolates of clusters A–C: 21 of the 27 analyzed virulence-associated genes were detected in all isolates (Figure 2). However, antimicrobial resistance genes (ARGs), were detected exclusively in isolates of cluster B (Figure 2). Three isolates carried genes conferring resistance to aminoglycosides (*aph(3*″*)-Ib*), chloramphenicol (*catA3*), sulfonamides (*sul2*) and tetracyclines (*tet*(B)), and two isolates carried genes conferring resistance to aminoglycosides (*aph*(*3*″)*-Ib*, *aph*(*3*″)*-Ia*, and *aph*(*6*)*-Id*), macrolides (*mef*(C) and *mph*(G)), sulfonamides (*sul2*), and tetracyclines (*tet*(H) and *tet*(Y)). One of these two isolates also carried *aaDA31*, conferring resistance to aminoglycosides (Figure 2). These last two strains harbored an integrative and conjugative element (ICE). No plasmids were detected using PlasmidFinder v2.0.1. 

### 3.4. Phylogenetic Relatedness, Virulence-Associated Genes, Mobile Genetic Elements, and Acquired ARGs of Respiratory Bovine P. multocida Isolates of Sequence Types ST13, ST79, and ST80

**Figure 3:**
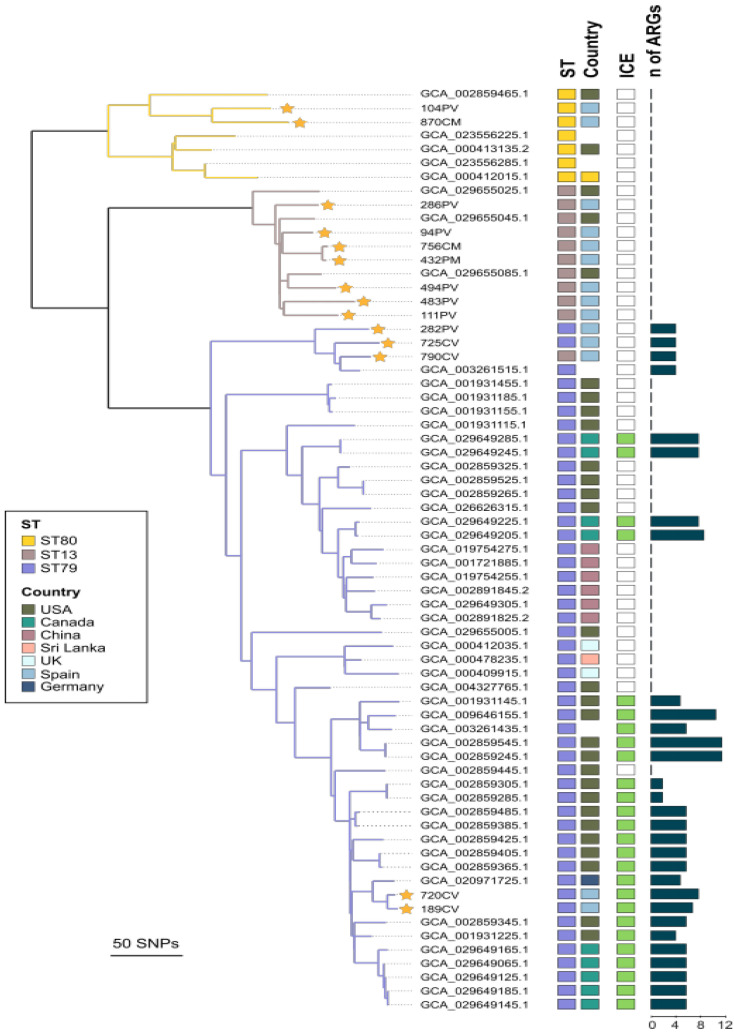
Phylogenetic tree generated on the basis of whole-genome SNP (WG-SNP) analysis of 67 bovine *P. multocida* isolates belonging to sequence types ST13, ST79, and ST80. These include 14 genomes sequenced in this study (indicated by a yellow star) and 53 genomes retrieved from the National Center for Biotechnology Information (NCBI) Pathogen Detection database. The figure also includes information about the country of origin, in silico identification of sequence type (ST), and the presence of integrative and conjugative elements (ICE) and antimicrobial resistance genes (ARGs). Colored lines of the phylogenetic tree indicate the main detected clusters. The scale ranging from 0 to 12 indicates the number of ARGs detected per isolate.

The combined WG-SNP phylogenetic analysis of the genomes of the 14 strains sequenced in this study and those of the genomes of the 53 strains retrieved from the NCBI Pathogen Detection database aligned with 91.8% of the reference ST79 genome (CP029322.1), identifying polymorphisms in 1576 sites. *P. multocida* genomes with the same MLST genotypes clustered together, except one isolate, ST13, which clustered with ST79, with a maximum difference of 315 SNPs between the ST13 and ST79 clusters, 367 SNPs between the ST80 and ST79 clusters, and 343 between the ST13 and ST80 clusters (Figure 3). Consistent with the results obtained for the 14 *P. multocida* strains sequenced in this study, no differences in the content of VAGs were detected in the genomes of the ST13, ST79, and ST80 strains retrieved from the NCBI Pathogen Detection database, and the vast majority (21 of 27) of VAGs were uniformly detected in strains of the three STs. The presence of different ARGs (between 2 and 12) was found exclusively in genomes of ST79 strains, with 53.1% of the strains of this ST carrying different ARGs (Figure 3). 

The detected ARGs conferred resistance to aminoglycosides, beta-lactams, lincosamides, macrolides, phenicols, streptogramins, sulfonamides, and tetracyclines (Table 2). No plasmids were detected using PlasmidFinder in any of the isolates. ICEs were detected in 86.7% of the ST79 strains harboring ARGs (Figure 3). Within the ST79 cluster, two isolates from this study that harbored ICE, 720CV and 189CV, had 10 SNP differences between them and a maximum of 27 SNP differences with the German bovine isolate (GCA_020971725.1) (Figure 3) that harbored ICE Tn*7407* [26]. By mapping the Illumina short-read data from the two studied isolates against the Tn*7407* ICE, a near-complete match was found, with an identity of 99.2% and 100% coverage of the ICE. 

## 4. Discussion

*P. multocida* is one of the main bacterial pathogens associated with BRD, mostly occurring in fattening animals [2,3,4]. This bacterium is also a common nasopharyngeal commensal able to descend and proliferate into the lungs following stress conditions or viral infections [4]. Several studies have characterized *P. multocida* isolates from cases of BRD [5,6,13,21,22], but studies focusing on the comparison of *P. multocida* isolates from cases of BRD with those from healthy animals are uncommon. Thus, this is the first study to compare the genetic characteristics of *P. multocida* isolates recovered from apparently healthy animals at the time of entry to the feedlot with isolates from animals affected by BRD during the fattening period through the use of different molecular techniques. 

As expected on the basis of previous data [5,13,15,22], all *P. multocida* isolates belonged to genotype A: L3, corroborating the association of this genotype with clinical cases of BRD, as well as the limited capacity of capsular LPS genotyping to show the genetic diversity of *P. multocida* isolates and to differentiate between related strains [5,8]. *P. multocida* isolates from BRD cases and those from healthy animals were further characterized by PFGE due to its high discriminatory power [32]. Isolates from healthy animals exhibited a relatively high genetic diversity (GD 0.60), which is consistent with the diversity of different *P. multocida* strains that can be identified in the respiratory tract of calves [33]. On the other hand, *P. multocida* isolates from calves affected by BRD exhibited a lower genetic diversity (GD 0.43), which is also consistent with previous data [5,6,33]. Most clinical isolates (81%) belonged to five pulsotypes that grouped into a single cluster, exhibiting at least 78% genetic similarity (Figure 1), supporting the close genetic relatedness of most *P. multocida* isolates associated with BRD [5,6,13]. 

Whole-genome sequencing (WGS) has been used for the genotyping of BRD-associated *P. multocida* isolates [15]. Thus, a subset of 14 *P. multocida* isolates representative of pulsotypes identified in isolates from both clinically BRD-affected calves and apparently healthy calves were selected for WGS, and in silico analysis of multilocus sequence types, virulence-associated genes, and antimicrobial resistance genes was carried out. The 14 *P. multocida* isolates belonged to sequence type ST80 but mainly to ST79 and ST13 (Figure 2), supporting findings that identified the latter two STs as the most frequently detected in a recent study that characterized *P. multocida* BRD-associated isolates in Spain [5]. The three STs were detected in isolates from clinical cases and isolates from apparently healthy calves, but ST13 was the most frequently found among isolates of healthy animals, and ST79 was the most frequent among isolates of clinical cases (Figure 2). This is consistent with the fact that ST79 is the most common sequence type detected worldwide among *P. multocida* isolates from BRD [6,13,14]. 

Phylogenetic analysis based on whole-genome SNP analysis grouped isolates of ST13, ST79, and ST80 into different genetically related clusters (Figure 2). Most ST13 isolates grouped into cluster A, and all but one were isolated from healthy animals, while cluster B included isolates from clinically affected animals of pulsotypes 1, 2, 4, and 5 belonging to PFGE cluster I and assigned to ST79 and one to ST13, indicating the close genetic relatedness of these pulsotypes. Isolates of pulsotypes 1 and 2 identified in this study exhibited indistinguishable restriction patterns from profiles B and A, respectively, which were the most frequently identified pulsotypes in BRD outbreaks that occurred on farms in various geographical areas in Spain [5], likely indicating their widespread distribution and their successful association with the disease. This success may be related to a higher occurrence in the upper respiratory tract compared to isolates of other pulsotypes and/or a higher capacity of these genotypes to cause infection in cattle [5]. Isolates of pulsotypes 1 and 2 were isolated from animals in only half of the batches included in the study and represented approximately 15% of the *P. multocida* isolates from healthy calves (Figure 1). The frequency of detection of these pulsotypes could have been higher if more animals from the batches had been sampled at the time of entry to the feedlot or if more colonies with characteristics compatible with *P. multocida* had been identified. Nevertheless, it is doubtful that an increased presence of these genotypes in the respiratory tract of healthy animals at the time of entry to the feedlot could explain their high frequency in *P. multocida* isolates from clinical cases.

*P. multocida* contains many genes encoding putative virulence factors that contribute to its pathogenesis [8]. In the present study, 27 different virulence-associated genes encoding fimbriae and other adhesins, toxins, iron acquisition proteins, sialic acid metabolism, outer membrane proteins (OMPs), and superoxide dismutase (Table S2) were screened to obtain comprehensive data on the prevalence of these genes in *P. multocida* isolates of the three identified STs. We did not detect differences in the virulence-associated gene contents in the 14 *P. multocida* genomes that could favor the colonization of the respiratory tract or host–pathogen interactions, regardless of the ST and the clinical or non-clinical status of the animals (Figure 2), making those pulsotypes more capable of producing infection. Differences in the expression of certain virulence-associated genes have been associated with a higher virulence of *P. multocida* BRD isolates [34]. Researchers explored the virulence difference mechanism of a naturally occurring attenuated strain and a highly virulent strain isolated from calves using transcriptome sequencing analysis, finding that the expression of several virulence-related genes, mainly capsule, iron utilization, lipopolysaccharide, and OMP-related genes, was upregulated in infection with the highly virulent strain compared to infection with the attenuated strain. These data indicate that for improved knowledge of the pathogenesis of *P. multocida* BRD infections, more attention should be paid to transcriptomic analysis to determine the level of gene expression rather than to merely genomic analysis to detect the presence or absence of virulence genes. The fact that no transcriptomic analyses were carried out to evaluate the expression of these virulence-associated genes represents a limitation of this study. 

The most significant difference found in this study between isolates of cluster B, mainly ST79 clinical isolates, and isolates of the two other clusters, was the detection of several ARGs (Figure 2; Table 2), which confer resistance to aminoglycosides, chloramphenicol, sulfonamides, tetracycline, and macrolides, the latter two of which are commonly used for the treatment of BRD [35].

As the results of this study may be biased by the relatively low number of feedlots included and their narrow geographical distribution, the genomes of the 14 *P. multocida* isolates sequenced in this study were further compared with the genome sequences of 53 *P. multocida* bovine isolates of ST13, ST79, and ST80 from different geographical locations available in the NCBI genome database (Table S1). These isolates originated from seven countries on three continents, thereby enabling a global comparison with the isolates investigated in this study. The results obtained from the global analysis of the genomes supported those obtained with the genomes of the 14 strains sequenced in this study. Thus, the phylogenetic analysis confirmed the clustering of the strains of each ST in separate but closely genetically related clades (Figure 3), as can be deduced from the fact that the maximum difference among the 67 genomes of *P. multocida* was 367 SNPs using ST79 genome CP029322.1 as a reference. Similarly, the virulence-associated genes were also constantly present in *P. multocida*, regardless of the ST of the isolates. As information on the clinical status of the animals for the vast majority of *P. multocida* isolates whose genomes were retrieved from the NCBI genome database was not available, we were not able to analyze possible differences in the contents of virulence-associated genes between clinical isolates and isolates from apparently healthy animals within the same ST. 

In accordance with the results observed in the genomes of the 14 isolates sequenced in this study, the main difference among isolates of the three STs was the detection of ARGs exclusively in the genomes of ST79 strains, which were associated, in most cases, with the presence of a putative ICE (Figure 3). ICEs are a class of mobile genetic elements and often function as vehicles for the spread of antimicrobial resistance factors [25,26]. *ICEPmu1*, harboring 12 different ARGs, is the ICE element most commonly detected in ST79 *P. multocida* isolates of bovine origin in North America [14]. Recently, a new ICE, *Tn7407*, which carries five ARGs that confer resistance to aminoglycosides (*aph*(*3*″)*-Ib*, *aph*(*3*″)*-Ia*, and *aph*(*6*)*-Id*), sulfonamides (*sul2*), and tetracycline (*tet(H*), and is genetically related to *ICEPmu1*, was described in a German isolate from a fatal case of BRD [26]. Since both ST79 Spanish isolates carrying an ICE element (720CV and 189CV; Figure 3) were closely related (27 SNP maximum difference) to the German isolate (accession number GCA_020971725.1), we mapped their sequences against the sequence of *Tn7407* detected in the German isolate. Both Spanish isolates showed a 99.2% identity with a 100% coverage with the ICE Tn*7407* and also carried the same five ARGs detected in Tn*7407*. In addition to the ARGs carried in putative ICE Tn*7407*, both Spanish isolates harbored three genes, (*mef(C)* and *mph(G)*), and *tet(Y)*)*,* coding resistance for macrolides and tetracycline. The short-read sequencing used in this study for the characterization of mobile elements such as ICEs often struggles to fully resolve their structures due to the complex and repetitive nature of these elements. Thus, we could not determine the precise genome location of these ARGs, and future studies using long-read sequencing technologies are necessary to provide a more comprehensive characterization of ICEs in these isolates. The two Spanish isolates were obtained from animals from two different batches (LV1 and LV8) with entry to the feedlot within 8 months each other (Table 1) and from different geographical origins; this might suggest that the presence of Tn*7407* among Spanish ST79 isolates could be more disseminated than may be concluded from the present study. 

To date, ICE Tn*7407* has been identified in a limited number of isolates in two European countries, but this result leaves open the possibility that the antimicrobial resistance in ST79 *P. multocida* isolates in Europe could be associated with a different ICE than that commonly detected in North American isolates. Further studies including a larger number of ST79 isolates from different European countries are necessary to confirm this possibility. The common detection of ARGs in *P. multocida* isolates that confer resistance to several antimicrobials commonly used for the treatment of BRD represents a risk by limiting therapeutic options, as the effectiveness of antimicrobial treatments can be compromised by the increasing occurrence of resistance in infecting strains [36]. In this sense, the detection of distinct ICEs carrying ARGs in ST79 isolates in different countries may represent a driving force favoring the selection and further spread of this epidemic linage associated with BRD cases. This spread might also be favored by the movement of animals, as the movement and introduction of stocks in farms appear to be relevant factors in the epidemiology of *P. multocida* [6]. Consistent with this idea, the ST79 isolates identified in this study were detected only in those feedlots that purchased animals of different origins for fattening. 

## 5. Conclusions

The genome analysis of *P. multocida* isolates from clinically and non-clinically affected calves did not detect differences in the content of virulence-associated genes that could explain the higher frequency of detection of particular clones in cases of BRD and between these clinical isolates and those isolated from healthy animals. The most significant finding was the detection in clones commonly found in BRD cases, such as ST79, of ARGs coding for resistance to different antimicrobial agents classically used for the treatment of BRD bacterial infections; this may have contributed to their selection and further dissemination between herds, mainly associated with the movement and mixing of animals of different origins.

## Figures and Tables

**Table 1:** Feedlots and batches followed-up during the fattening period (six to seven months) in this study.

Feedlot	Feedlot Location	Batch	Feedlot Entry Date	Animal Source	No. of Animals
1	Valencia	LV1	13 April 2021	Purchased	80
		LV2	15 June 2021	Purchased	87
		LV3	15 September 2021	Purchased	80
		LV4	2 November 2021	Purchased	83
		LV5	5 May 2022	Purchased	80
		LV6	14 June 2022	Purchased	85
		LV7	13 January 2022	Purchased	80
		LV8	26 January 2022	Purchased	75
		LV9	18 February 2022	Purchased	74
		LV10	24 March 2022	Purchased	87
		LV11	31 March 2022	Purchased	85
		LV12	9 June 2022	Purchased	98
2	Madrid	LM1	18 January 2021	Own farm	26
		LM2	11 April 2021	Own farm	24
		LM3	25 May 2021	Own farm	28
3	Madrid	LM4	10 October 2021	Own farm	30
4	Madrid	LM5	20 September 2022	Purchased	28
5	Madrid	LM6	23 May 2022	Own farm	20
6	Madrid	LM7	11 May 2022	Own farm	14
7	Madrid	LM8	12 May 2022	Own farm	18

**Table 2:** Frequency of antimicrobial resistance genes by drug class among the 67 bovine *P. multocida* genomes belonging to sequence types ST13, ST79, and ST80.

Class	No. of Isolates	%	Genes
Aminoglycosides	30	44.7	*aadA25*; *aadA31*; *ant*(*2*″)*-Ia; aph*(*3*″)*-Ib*; *aph*(*3*′)*-Ia*; *aph*(*6*)*-Id*
Beta-lactams	4	5.9	*bla*_OXA-2_
Lincosamides	5	7.0	*erm42*
Macrolides	11	16.4	*mefC*; *mphE*; *msrE*; *erm42*
Phenicols	10	14.9	*catA3*; *floR*; *msrE*
Streptogramins	9	13.4	*msrE*; *erm42*
Sulfonamides	25	37.3	*sul2*
Tetracyclines	30	44.7	*tetB*; *tetH*; *tetY*

## Data Availability

The data used in this study can be found in the article’s Supplementary Materials (Table S1). The genomic sequencing data generated throughout this research have been deposited under project PRJNA988320, publicly available in the National Center for Biotechnology Information (NCBI) Database.

## Supplementary Materials

The following supporting information can be downloaded at: https://www.mdpi.com/article/10.3390/ani13172687/s1, Table S1: Bovine *Pasteurella multocida* strains used in this study; Table S2: Virulence-associated genes (VAGs) searched in the 67 bovine *P. multocida* genomes belonging to ST13, ST79, and ST80.
