# Supplementary material for: Genotypic Comparison of Pasteurella multocida from Healthy Animals at Entry to the Feedlots with That and from Bovine Respiratory Disease-Affected Animals during the Fattening Period
Source: Animals (Basel). 2023 Aug 22;13(17):2687. doi: 10.3390/ani13172687 (PMC10487216; doi:10.3390/ani13172687)
Supplement: Supplementary file 1 [file animals-13-02687-s001.zip › Table S2.pdf]

Table S2. Virulence associated genes (VAGs) searched in the 67 bovine *P. multocida* genomes belonging to ST13, ST79 and ST80.

Function	VAGs	Accession number	Description
**Adhesins**	*hsf1*	AAK02798.1	Autotransporter adhesion
	hsf2	ANJ91000.1	Autotransporter adhesion
	flp1	AFF24176.1	Flp pilin
	ptfA	AKO69808.1	Type 4 fimbriae
	fimA	AAK03010.1	Fimbriae
	pfhA	AAK61595.1	Filamentous hemagglutinin
	pfhB1	AAK02141.1	Filamentous hemagglutinin
	tadD	AAK02930.1	Putative nonspecific tight adherence protein D
**Iron acquisition factors**	*exbB*	AAK03270.1	Accessory protein Ton-dependent transport of iron compounds
	exbD	AAK03271.1	Accessory protein Ton-dependent transport of iron compound
	fur	AAK02436.1	Ferric uptake regulation protein
	hgbA	AAQ14873.1	A hemoglobin-binding protein
	hgbB	APX53047.1	B hemoglobin-iron uptake
	tbpA	AAK02460.1	Transferrin binding protein A
	tbpB	AAX55324.1	Transferrin binding protein B
	tonB	AAK03272.1	Iron transporters, transport ferric-siderophore complexes
**Outer membrane proteins**	*plpB*	AAK03814.1	Lipoprotein B
	plpE	AAK03601.1	Lipoprotein E
	ompA	AFU91511.1	Outer membrane protein A
	ompH	AAK02472.1	Outer membrane protein H
	OMA87	AAC44600.1	Outer membrane protein 87
**Hyaluronidase**	*PmHAS*	AAC38318.2	Hyaluronan synthase
**Sialidases**	*nanB*	AAG35309.1	Outer membrane-associated proteins, an autotransporter protein
	nanH	AAG35310.1	Outer membrane-associated proteins, small sialidases
**Superoxid dismutases**	*sodA*	AAK02085.1	Superoxide dismutase
	sodC	AAK04036.1	Superoxide dismutase
**Toxin**	*toxA*	AAW57319.1	Dermonecrotic toxin
